# Nano-TiO_2_ Is Not Phytotoxic As Revealed by the Oilseed Rape Growth and Photosynthetic Apparatus Ultra-Structural Response

**DOI:** 10.1371/journal.pone.0143885

**Published:** 2015-12-01

**Authors:** Jun Li, Muhammad Shahbaz Naeem, Xiuping Wang, Lixin Liu, Chang Chen, Ni Ma, Chunlei Zhang

**Affiliations:** 1 Oil Crops Research Institute, Chinese Academy of Agricultural Sciences, Wuhan, PR China; 2 College of Life Science and Technology, Hebei Normal University of Science and Technology, Qinhuangdao, PR China; 3 Department of Agronomy, University of Agriculture, Faisalabad, Pakistan; Huazhong university of Science and Technology, CHINA

## Abstract

Recently nano-materials are widely used but they have shown contrasting effects on human and plant life. Keeping in view the contrasting results, the present study has evaluated plant growth response, antioxidant system activity and photosynthetic apparatus physiological and ultrastructural changes in *Brassica napus* L. plants grown under a wide range (0, 500, 2500, 4000 mg/l) of nano-TiO_2_ in a pot experiment. Nano-TiO_2_ has significantly improved the morphological and physiological indices of oilseed rape plants under our experimental conditions. All the parameters i-e morphological (root length, plant height, fresh biomass), physiological (photosynthetic gas exchange, chlorophyll content, nitrate reductase activity) and antioxidant system (Superoxide dismutase, SOD; Guaiacol peroxidase, POD; Catalase, CAT) recorded have shown improvement in their performance by following nano-TiO_2_ dose-dependent manner. No significant chloroplast ultra-structural changes were observed. Transmission electron microscopic images have shown that intact & typical grana and stroma thylakoid membranes were in the chloroplast, which suggest that nano-TiO_2_ has not induced the stressful environment within chloroplast. Finally, it is suggested that, nano-TiO_2_ have growth promoting effect on oilseed rape plants.

## Introduction

Use of nanomaterials is one of the rapidly growing research areas during the last decade [1]. Nanomaterials are being applied in almost every field like cosmetics, medicine and agriculture etc. [2]. Nanomaterial’s (NMs) have tremendous potential to generate new ways to manipulate genome, DNA delivery and growth regulation in plants [3,4]. There is an extensive interest to investigate applying NMs to plants for agricultural use.

Nano-TiO_2_ is diversely used nanoparticles. Nano-TiO_2_ materials are being utilized as a disinfectant, antibiotic, biological sensor, tumor killing agent and antibacterial products [5]. Contrasting effects of nano-TiO_2_ on plant growth have been reported. Some studies have reported that nano-TiO_2_ is cytotoxic [6] but others are showing opposite results [7]. One study has shown that nano-TiO_2_ application may induce aged seeds vigor and chlorophyll content in spinach [8] and other has shown that nano-TiO_2_ is toxic for seed germination and root growth [9,10] which are considered as the most important basic toxicity research tools for plants. Overall, few systematic studies have been conducted to determine the effects of nano-TiO_2_ on plant physiology and plant development at the organism level. Information available about nano-TiO_2_ effect on chloroplast ultra-structural changes is scarce. However, literature has suggested that any abiotic change in the plant environment induce oxidative stress which in turn may damage the membrane system especially mitochondria and chloroplast ultra-structures [11] and ultimately hampers physiological performance of photosynthetic apparatus in plants. Limited literature is available on the plant biological and physiological effects of nano-TiO_2_ for its practical application in agriculture. Therefore, it is imperative to continue such studies to understand the effects of nano-TiO_2_ on plant growth and physiology.

Oilseed rape is considered as one of the main source of edible oil not only in China but all over the world. [12]. Therefore, oilseed rape potential must be exploited against various environmental stresses like nano-TiO_2_. Keeping in mind the importance of oilseed rape and contrasting biological responses of plants to nano-TiO_2_ toxicity, the present study was planned.

The study was executed with a wide range of nano-TiO_2_ toxicity on plant growth, antioxidant system and photosynthetic apparatus especially the chloroplast ultra-structures. Results from this research may help to understand the effects of nano-TiO_2_ in oilseed rape plants and further their application in the laboratory or field.

## Materials and Methods

### Characteristics of nanoparticles

Nano-TiO_2_ was purchased from the Shanghai Chemical Co. of China. Properties of the nano-TiO_2_ were as follows: aerosol, purity≥99.5%, anatase/rutile = 80:20 and particle size = 27 nm.

### Plant material and treatment conditions

Seeds of *Brassica napus* L. (cv. Zhongshuang No. 11) were purchased during August, 2014 from a local seed company-Wuhan Zhongnongyou Seeds Technologies Co., Ltd., Wuhan, Hubei Province, China (30°69N, 114°19E). The seeds were vernalized for 2 weeks and were sterilized for 10 min in 10% sodium hypochlorite solution before use.

Healthy seeds of *Brassica napus* L. (cv. Zhongshuang No. 11) were sown in plastic pots (30 cm diameter) having commercial soil (Sunshine Mix #5, Sun Gro, Canada). Five uniform plants per pot were allowed to grow after three weeks and there were four replications for each treatment. Forty four days old seedlings were sprayed with water or different concentrations of nano-TiO_2_ suspensions (500, 2500 and 4000 mg/l). Data for different parameters were recorded from the next day for four times with an interval of a week. Left over plants for each treatment were allowed to grow till maturity and then harvested to record the data for yield and yield components.

### Morphological parameters

Five plants for each treatment were randomly sampled next day after nano-TiO_2_ treatment to record the data for taproot length, plant height and biomass of the seedlings and this action was repeated four times with an interval of a week.

### Antioxidant enzymes assay

Crude enzyme was extracted from topmost fully expanded fresh leaf samples (0.5 g) by using potassium phosphate buffer with the help of mortar and pestle chilled at 4°C as described by Naeem et al. [13]. Extracted enzyme was used to determine the activity of following antioxidant enzymes:

Activities of catalase (CAT, EC1.11.1.6), superoxide dismutase (SOD, EC1.15.1.1) and guaiacol peroxidase (POD, EC1.11.1.7) were assayed following the protocols of Aebi [14], Zhou et al. [15], Zhou and Leul [16], respectively.

### Nitrate reductase assay and chlorophyll pigment

Assay was performed by extracting oilseed rape leaves with an extraction buffer comprised of Tris–HCl (250 mM) with pH 8.0, EDTA (1 mM), Na_2_MoO_4_ (1 μM), flavin adenine dinucleotide (5 μM), dithiothreitol (3 mM), BSA (1%), β-mercaptoethanol (12 mM) and PMSF (250 μM). After extraction, samples were centrifuged at 13000 rpm just for 5 minutes to achieve the supernatants which in turn were mixed with a buffer having NaNO_3_-40 mM, Na_2_HPO_4_-80 mM, NaH_2_PO_4_ (pH 7.5)-20 mM and NADH-0.2 mM. At 25°C after 2 hours incubation, sulphanilamide-1% and N-(1-napthyl) ethylenediamine hydrochloride-0.05% were added to cease the reaction and finally absorbance was recorded with the help of spectrophotometer at 540 nm to calculate the concentration of nitrite [17].

Chlorophyll was extracted from the leaves by soaking in acetone and alcohol (1:1) mixture solution. Total chlorophyll contents were spectrophotometrically recorded [18].

### Transmission electron microscopy

Completely unfolded leaves at the top of plants were used to obtain the control and treated samples excluding veins. Samples were washed thrice with glutaraldehyde-4% in phosphate buffer-0.1M after treatment with the same buffer for more than 12 hours. After incubation for 1 h in OsO_4_-1%, samples washing were repeated thrice after every ten minutes. In the next step, samples were dehydrated using gradually increased concentration of ethanol from 50–100% and ultimately for twenty minutes with acetone. After infiltration and embedding with Spurr’s resin, samples were heated at 70°C for nine hours. Finally, transmission electron microscope (JEOL TEM-1230EX) was used to observe the ultra-structures of the samples on copper grids [13].

### Photosynthetic gas exchange

Photosynthetic gas exchange characteristics of healthy leaves were recorded at 10: 00 am in the morning with a CIRAS-1 portable photosynthesis system (PP-Systems, UK). Randomly three healthy and functional leaves were selected for each measurement. Photosynthetic parameters like net photosynthetic rate (*Pn*), stomatal conductance (*Gs*), intercellular CO_2_ concentration (*Ci*) and transpiration rate (*Tr*) were recorded and replicated at least eight times [19].

### Statistical analysis

Analysis of variance was performed with statistical package prism 5.0. Data means were subjected to Student′s t test for comparison at p<0.05.

## Results

### Plant growth response

**Fig 1:**
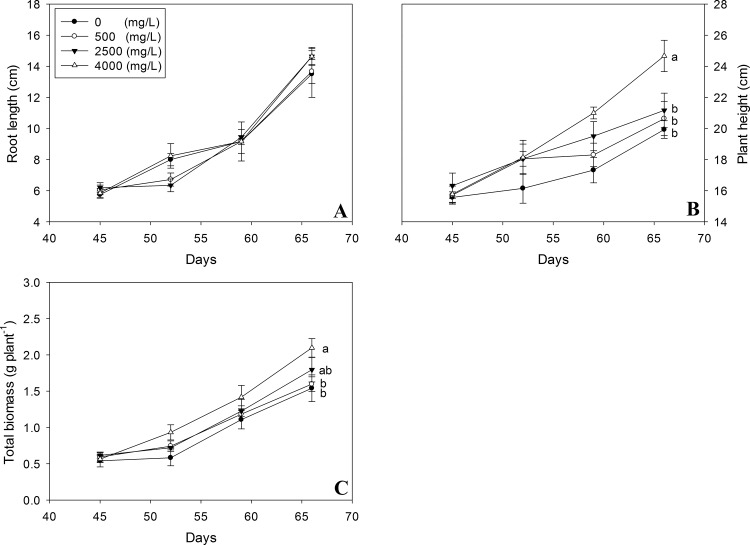
Effects of nano-TiO_2_ on seedling growth of oilseed rape. Results are shown as an average of measurements for five plants. Means with the same lowercase letters are not significantly different at P<0.05. Vertical bars represent±SE.

Phytotoxic effects of nano-TiO_2_ were recorded on plant growth in terms of taproot length, plant height and fresh biomass (Fig 1). A positive but not significant change was recorded for taproot length of oilseed rape plants (Fig 1A). However, oilseed rape plants height was increased after treated with different concentrations of nano-TiO_2_ (Fig 1B). Maximum height was recorded with 4000 mg/l nano-TiO_2_. The total biomass of plant vegetation (leaves, stems, and roots) of nano-TiO_2_-exposed seedlings increased approximately by 30–40% compared with control seedlings (Fig 1C). Increase in the biomass followed the dose dependent pattern of nano-TiO_2_. Minimum biomass was recorded in control plants and maximum at 4000 mg/l nano-TiO_2_ treated plants.

### Antioxidant enzymes activity

**Fig 2:**
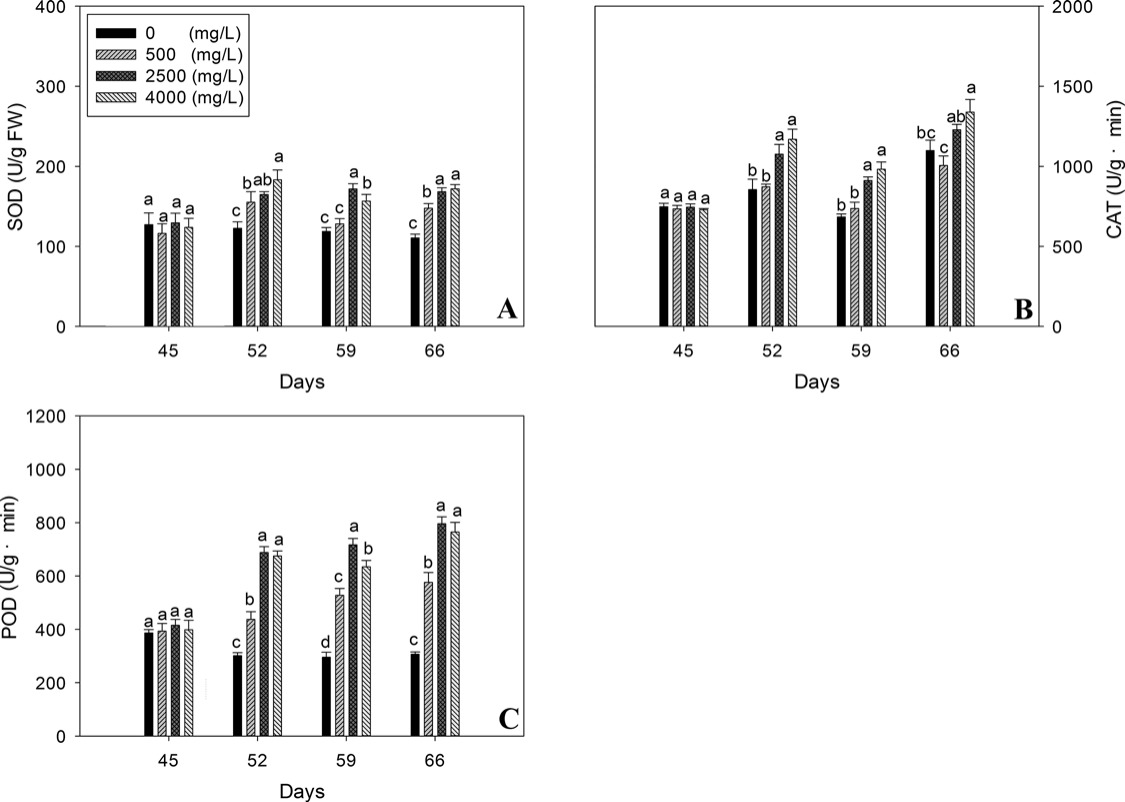
Effects of nano-TiO_2_ on antioxidant enzymes activity of oilseed rape. Results are shown as means of four replicates. Means with the same lowercase letters are not significantly different at P<0.05. Vertical bars represent±SE.

The effect of nano-TiO_2_ on the protective antioxidant enzymes activity such as SOD**,** CAT and POD of oilseed rape is shown in Fig 2. Results have shown that immediately after exposure to nano-TiO_2_ at 45 days, the SOD, CAT and POD activity of oilseed rape depicted no significant change with the increase of nano-TiO_2_ as compared to control plants. However, during next three weeks (52, 59 and 66^th^ day) SOD, CAT and POD activity of *B*. *napus* was significantly increased compared to the respective controls and followed the dose dependent pattern of nano-TiO_2_ (Fig 2A–2C). Maximum activities for all the enzymes tested were recorded in 66 days old plants treated with 4000 mg/l nano-TiO_2_. Overall, enzymes results have shown that the effect of nano-TiO_2_ on the activity of protective enzymes follow the same trend.

### Nitrate reductase activity and chlorophyll content

**Fig 3:**
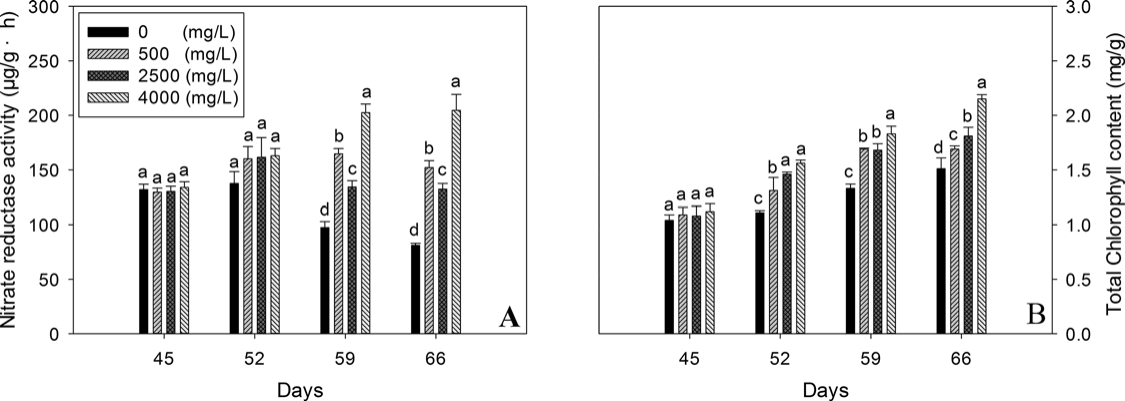
Nitrate reductase activity and Chlorophyll content of oilseed rape leaves at different days after exposure to nano-TiO_2_. Results are shown as means of four replicates. Means with the same lowercase letters are not significantly different at P<0.05. Vertical bars represent±SE.

Effect of nano-TiO_2_ on nitrate reductase activity and chlorophyll content is shown in the Fig 3. Results have shown that increasing dosage of nano-TiO_2_ has not induced significant change in the NR at 45^th^ & 52^nd^ day old seedlings. However, later on the activity of NR increased significantly and followed the concentration dependent pattern. Maximum values of NR were recorded at 66^th^ day (Fig 3A).

Chlorophyll content data recorded for 45 days old seedlings showed no significant change with the increase of nano-TiO_2_ dosage (Fig 3B). However, the chlorophyll content of oilseed rape seedlings during the next weeks were dramatically higher and followed the nano-TiO_2_ concentration dependent pattern with maximum chlorophyll content in 66 days old plants treated with 4000 mg/l nano-TiO_2_. These results have shown that nano-TiO_2_ can significantly increase chlorophyll content of oilseed rape.

### Photosynthetic apparatus physiological performance

**Fig 4:**
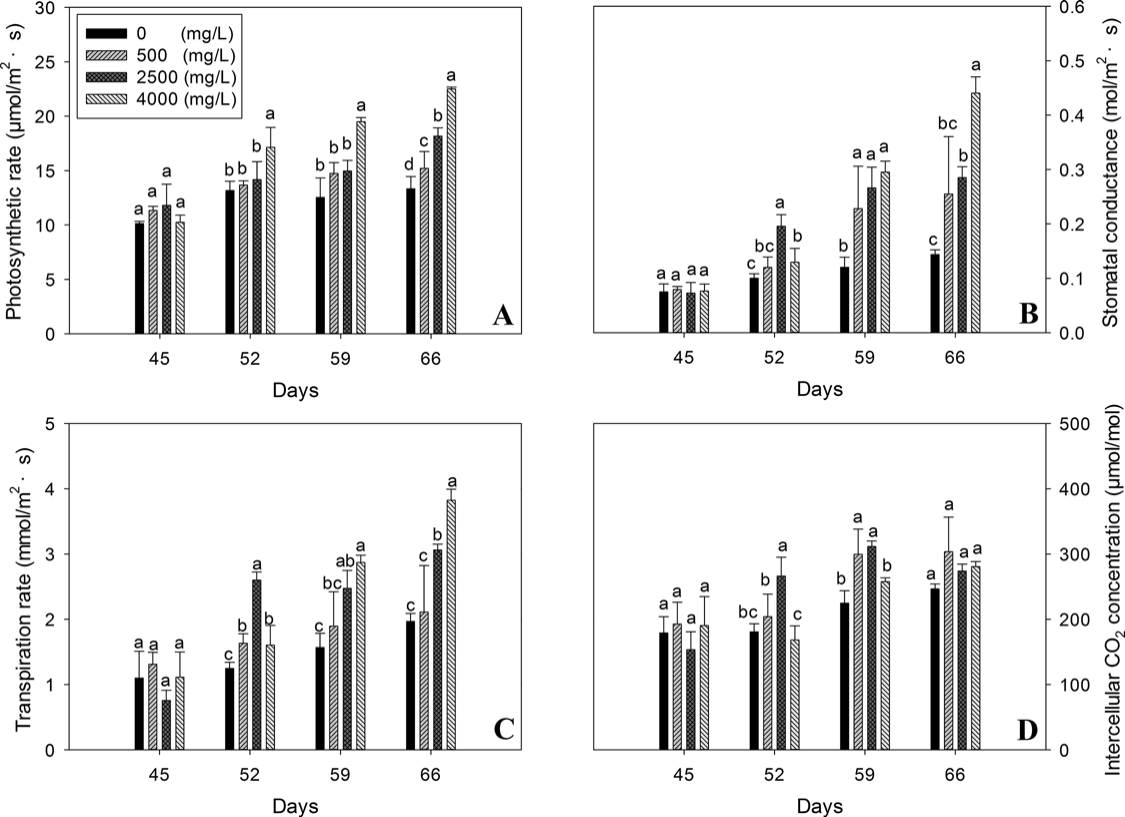
Photosynthetic apparatus performance of oilseed rape plants at different days after exposure to nano-TiO_2_. A: net photosynthetic rate (Pn); B: stomatal conductance (Gs); C: intercellular CO_2_ concentration (Ci); D: transpiration rate (Tr). Results are shown as means of four replicates. Means with the same lowercase letters are not significantly different at P<0.05. Vertical bars represent±SE.

Fig 4 shows the photosynthetic apparatus physiological changes induced by nano-TiO_2_ in oilseed rape plants. Photosynthetic parameters, i.e. net photosynthetic rate, stomatal conductance, internal CO_2_ concentration and transpiration rate showed no significant change just after the exposure to nano-TiO_2_ at 45 days old seedlings. However, a significant increase was recorded with the increase in TiO_2_ concentration during the coming weeks and generally this increase was gradual. Maximum performance for all these parameters was recorded at 66^th^ day of seedling age with 4000mg/l nano-TiO_2_ concentration except intercellular CO_2_ concentration whose results were indifferent.

### Ultra-structural changes in chloroplasts

**Fig 5:**
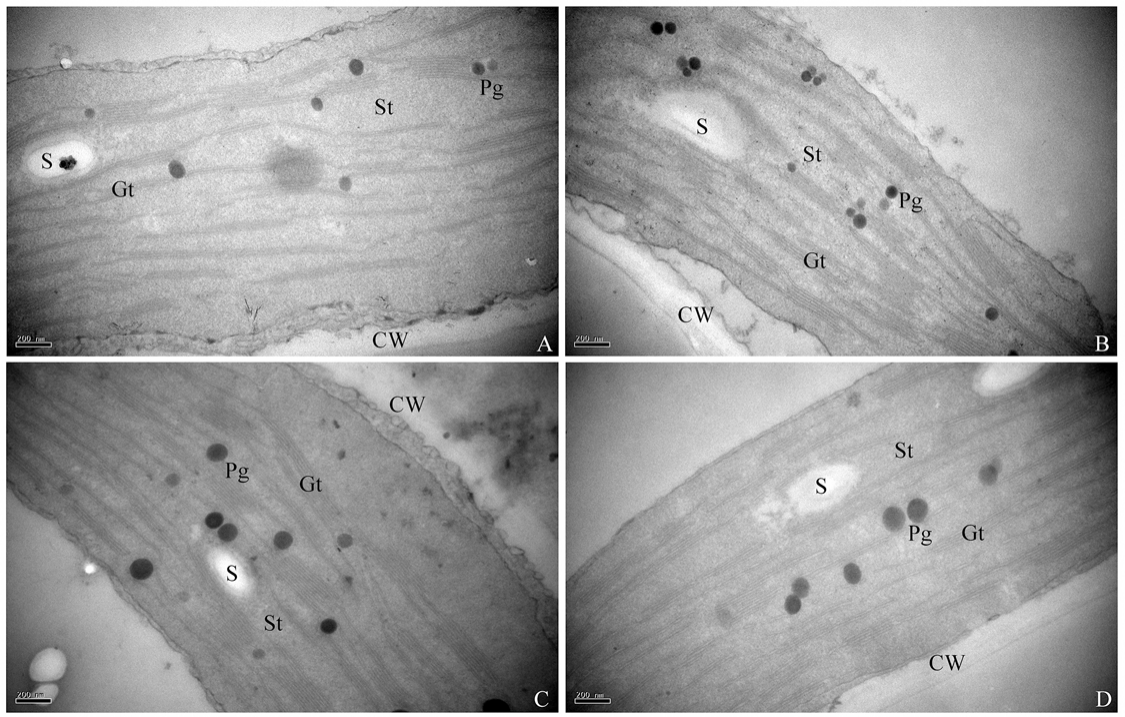
TEM images of the chloroplast ultra-structures at 66 day old oilseed rape seedlings. Chloroplast ultra-structures (A) control (B) nano-TiO_2_ @ 500mg/l (C) nano-TiO_2_ @ 2500mg/l (D) nano-TiO_2_ @ 4000mg/l. CW, cell wall; St, stroma thylakoids; Gt, grana thylakoids; S, sugar grains; Pg, plastoglobuli. Bars A-B = 200 nm.

Thylakoid membrane system was observed typical with no drastic changes in nano-TiO_2_ treated chloroplasts compared to the control. Grana and stroma membrane stacks were intact and there was no swelling in the stroma when treated with nano-TiO_2_. Starch grains were present in the chloroplast and more importantly plastoglobuli were lesser in number especially in 4000 mg/l nano-TiO_2_ treated plants compared to the control plants. Generally speaking, an increase in the dosage of nano-TiO_2_ didn’t induce any negative change in chloroplast ultra-structures (Fig 5A–5D).

### Effect on yield and yield components

**Fig 6:**
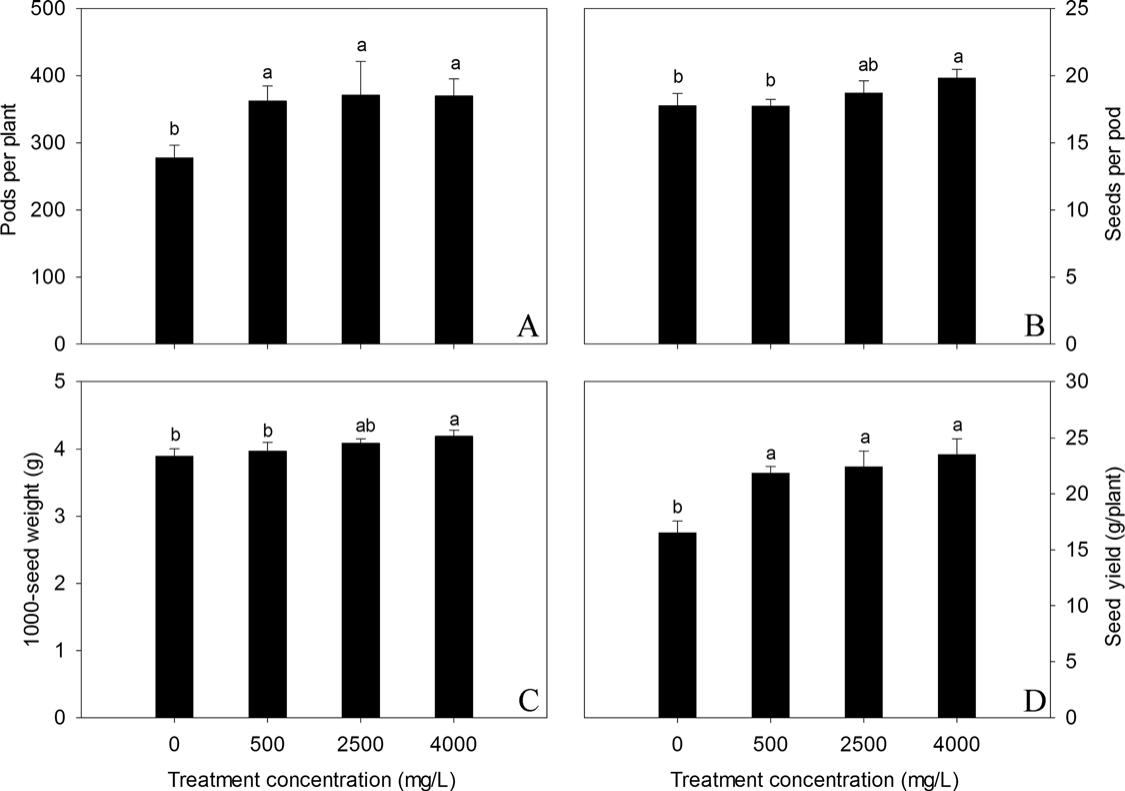
Effects of nano-TiO_2_ on yield and yield components of oilseed rape. Results are shown as means of four replicates. Means with the same lowercase letters are not significantly different at P<0.05. Vertical bars represent±SE.

At maturity, effect of nano-TiO_2_ on yield and yield components was monitored in terms of pods per plant, seeds per pod, 1000-seed weight and seed yield as shown in Fig 6. There was no statistical significant change for number of seeds per pod (Fig 6A). However, 1000-seed weight, seed yield per plant and number of seeds per pod was improved by increasing dose of nano-TiO_2_ especially at higher dose of 4000 mg/l (Fig 6B–6D). Generally speaking, results are showing that nano-TiO_2_ have no toxic effect on the ultimate seed yield of oilseed rape plants rather improves it.

## Discussion

Nano-toxicity to plant life is an emerging arena and is being focused in the recent years [20]. Contrasting effects of nano-materials on plant growth and development is a feature of the previous studies. The present study is an effort to understand how diverse range of nano-TiO_2_ impacts the plant growth and development in oilseed rape.

Enhanced root length, plant height, biomass in the present study indicates that nano-TiO_2_ might have induced the absorption of water and fertilizer [21]. Increase in growth parameters also demonstrates that nano-TiO_2_ has catalyzed the photosynthetic process as shown in Fig 4. This promotion in plant growth may also be due to the increased inorganic nitrogen (such as NO_3_^-^-N and NH^+^-N) conversion into organic nitrogen i-e protein and chlorophyll, which ultimately improve the plant growth [22, 23]. It could be speculated that relatively higher nitrate reductase activity (Fig 3A) in our present study might have provided the pool of NH_4_^+^ nitrogen by transforming the NO_3_^-^ to NH_4_^+^ in vivo during nitrogen metabolism and then accelerate the formation of chlorophyll (Fig 3B) which ultimately has induced the plant growth [24]. Nitrate reductase activity enhanced in the present study might be linked with nitrate absorption as it has been reported that nitrate reductase activity induced under nitrate [25]. Results of this study are consistent with the findings of all the tested plant species i-e *Brassica compestris* L., *Lactuca sativa* L. and *Phaseolus vulgaris* L. for root length [26] and spinach for biomass [27].

Although nano-TiO_2_ is not phytotoxic as revealed by the performance of plant growth indices but it has activated the antioxidant system (Fig 2) of oilseed rape plants as a matter of defense in response to oxidative stress [28, 25]. This means that abiotic stress was induced by nano-TiO_2_. Dose dependent increase in enzymes activities suggest us that increasing dose of nano-TiO_2_ would have increased the production of reactive oxygen species (ROS) and in turn antioxidant system enzymes activities were activated in the same fashion [29, 30]. Enhanced antioxidants activities suggest that nano-TiO_2_ induced stress was not severe to destroy the antioxidant system apparatus in the plants, rather activated it as a matter of defense and ultimately overall growth of plants.

Plant growth response and grain yield is ultimately controlled by the production of photosynthates. Induced photosynthetic gas exchange capacity by nano-TiO_2_ by following the dose dependent manner suggest that nano-TiO_2_ might has increased the absorption of nitrogen and magnesium minerals to promote the chlorophyllase activity and hence the chlorophyll synthesis which in turn might have increased light absorbance, improved light energy traffic and ultimately has avoided the chloroplasts damage (Fig 5) and prolonged the photosynthesis time of chloroplasts [31].

To analyze the chloroplast damage, transmission electron microscopic chloroplast ultra-structural images were executed which have revealed intact grana-stacks and no swelling in stroma (Fig 5) which means that either there was no over-production of ROS in the chloroplast or scavenged by the antioxidant system activated (Fig 2) by nano-TiO_2._ Chloroplast serves as an apparatus for photosynthesis reactions [32]. Reports have shown that chloroplast ultra-structures are affected by toxicity and in turn hamper the photosynthesis [33]. Under stress conditions, transpiration is hampered due to stomata closure, which declines the CO_2_ concentration within chloroplasts and consequently affects NADPH^+^ production and let the ferredoxin electrons reduce O_2_, ultimately induces the formation of reactive oxygen species like H_2_O_2_, OH^-^ etc. [34]; these species may deteriorate the membrane system of the plant such as chloroplast. Few plastoglobuli observed in the chloroplast (Fig 5) is an indication of no lipid peroxidation of thylakoid or cell membrane. Presence of starch granules in the chloroplast also reveals that there was no stressful environment induced by the TiO_2_ in chloroplast, as complex sugars have not transformed into simple soluble sugars which are supposed to be the major compatible solutes for osmotic adjustment [35]. Improved yield and yield components data for our study is the ultimate response of nano-TiO_2_ on oilseed rape plants which confirms that nano-TiO_2_ is not toxic rather improves plant performance.

In summary, this article suggests that nano-TiO_2_ is non-phytotoxic as revealed by the improved photosynthetic apparatus physiological performance, no drastic changes in the thylakoid membranes, improvement in the plant growth and ultimately better yield of the oilseed rape plants. However, further studies are required to establish it, keeping in mind dose, exposure time, plant species and growth stage variability.
